# Aberrant Methylation Status of Tumour Suppressor Genes in Ovarian Cancer Tissue and Paired Plasma Samples

**DOI:** 10.3390/ijms20174119

**Published:** 2019-08-23

**Authors:** Dana Dvorská, Dušan Braný, Bálint Nagy, Marián Grendár, Robert Poka, Beáta Soltész, Marianna Jagelková, Katarína Zelinová, Zora Lasabová, Pavol Zubor, Zuzana Danková

**Affiliations:** 1 Division of Molecular Medicine, Biomedical Center Martin, Jessenius Faculty of Medicine in Martin, Comenius University in Bratislava, 036 01 Martin, Slovakia; 2 Department of Human Genetics, Faculty of Medicine, University of Debrecen, H-4032 Debrecen, Hungary; 3 Bioinformatic Unit, Biomedical Center Martin, Jessenius Faculty of Medicine in Martin, Comenius University in Bratislava, 036 01 Martin, Slovakia; 4 Institute of Obstetrics and Gynecology, Faculty of Medicine, University of Debrecen, H-4032 Debrecen, Hungary; 5 Department of Gynaecology and Obstetrics, Martin University Hospital, Jessenius Faculty of Medicine in Martin, Comenius University in Bratislava, 036 01 Martin, Slovakia; 6 Division of Oncology, Biomedical Center Martin, Jessenius Faculty of Medicine in Martin, Comenius University in Bratislava, 036 01 Martin, Slovakia

**Keywords:** liquid biopsy, pyrosequencing, ovarian cancer, *CDH1*, *PTEN*, *PAX1*, *RASSF1*, cfDNA

## Abstract

Ovarian cancer is a highly heterogeneous disease and its formation is affected by many epidemiological factors. It has typical lack of early signs and symptoms, and almost 70% of ovarian cancers are diagnosed in advanced stages. Robust, early and non-invasive ovarian cancer diagnosis will certainly be beneficial. Herein we analysed the regulatory sequence methylation profiles of the *RASSF1*, *PTEN*, *CDH1* and *PAX1* tumour suppressor genes by pyrosequencing in healthy, benign and malignant ovarian tissues, and corresponding plasma samples. We recorded statistically significant higher methylation levels (*p* < 0.05) in the *CDH1* and *PAX1* genes in malignant tissues than in controls (39.06 ± 18.78 versus 24.22 ± 6.93; 13.55 ± 10.65 versus 5.73 ± 2.19). Higher values in the *CDH1* gene were also found in plasma samples (22.25 ± 14.13 versus 46.42 ± 20.91). A similar methylation pattern with positive correlation between plasma and benign lesions was noted in the *CDH1* gene (*r* = 0.886, *p* = 0.019) and malignant lesions in the *PAX1* gene (*r* = 0.771, *p* < 0.001). The random forest algorithm combining methylation indices of all four genes and age determined 0.932 AUC (area under the receiver operating characteristic (ROC) curve) prediction power in the model classifying malignant lesions and controls. Our study results indicate the effects of methylation changes in ovarian cancer development and suggest that the *CDH1* gene is a potential candidate for non-invasive diagnosis of ovarian cancer.

## 1. Introduction

Ovarian cancer (OC) is one of the most lethal gynaecological cancerous diseases and the fifth leading cause of cancer-related death in women. Although it is globally diagnosed in almost 240,000 women annually and responsible for over 150,000 deaths each year [1], the incidence varies regionally and it is generally higher in both developed countries and caucasian women. The following OC rates in 100,000 women have been established: (1) Eastern Europe has 11.4 women diagnosed with OC in every 100,000, (2) the Central Europe and North America have over 8, (3) Central and South America have up to 6 and (4) the lowest rates are observed in Asia and Africa, at less than 3, usually [1,2]. It is also important that OC incidence has been slightly decreasing in developed countries since 1990 but increasing in developing countries [3,4,5]. OC is also very heterogeneous, with a variety of benign, borderline and malignant variants, almost all of which arise from transformation of epithelial, stromal and germ cell types [6]. Of these, the most common malignant neoplasms have their origin in the epithelium in up to 90% OC while the germ cell type are very rare with only 2–3% [6].

Ovarian tumours (OT) were traditionally divided into five groups: Serous, mucinous, endometrioid, clear cell and transitional [7]. However, each of those also comprised variable forms of benign, borderline and malignant lesions, with a total of over 40 tumour types. This classification primarily focused on the ovarian mesothelial surface as the point of origin for epithelial OT [7], but the more recent 2014 WHO classification, established in parallel with new FIGO staging implementation, excluded transitional cell tumours, but added the new sero-mucinous tumour group [7]. This latest classification is considered more consistent and it also comprises only 28 tumour types. While Meinhold-Heerlein et al. [7] have compared the traditional and new classification in detail many analyses have been based on older classification, and the results from those studies quoted herein are in their original form. However, it is essential to remember that OC is a very variable group of cancerous diseases and their formation is affected by many epidemiological factors [2]. It is especially typical in its lack of early signs and symptoms, and almost 70% of these cancers are diagnosed in advanced stages [8].

Diagnosed OC is primarily treated surgically with subsequent application of platine and taxane based adjuvant chemotherapy [9], but the efficacy of surgical treatment followed by chemotherapy rapidly decreases with the identified stage of the tumour [8]. The statistics for variable OC histotypes highlight that only 20% of women in advanced stages have five-year survival after diagnosis, while 89% diagnosed in stage I and 71% in stage II survive five years [8]. Moreover, OC metastases spread very quickly using two strategies: (1) Transcoelomic passive dissemination of tumour cell spheroids in the peritoneal cavity through ascites and (2) haematogenous metastasis of OC cells in the systemic circulation followed by the preferred seeding of the omentum [10,11,12]. It is therefore essential to use the robust, sensitive and non-invasive liquid biopsy (LB) approach for early OC diagnosis and differentiation. This methodology overcomes the typical limitations of solid tumour biopsies, such as invasiveness connected with unexpected complications during surgical procedures, difficulties in operating on organs that lie deep within the body, false negativity from sampling bias and the impossibility of repeating the same intervention [13]. Most importantly, solid biopsies provide no possibility for early tumour diagnosis and differentiation; they give tumour information only at particular points in time and they preclude monitoring the response to medication during treatment and follow up. In contrast, these are all possible with the LB approaches focused on detection and characterisation of abnormalities in circulating tumour cells (CTC), circulating tumour DNA (ctDNA), circulating cell-free microRNAs (cfmiRNA) and released exosome vesicles [10].

This cross-sectional study focuses on epigenetic changes, particularly alterations in methylation levels in primary tumours and the paired plasma samples. It then analyses the model’s predictive power in distinguishing between diagnoses so that this can be used in clinically predicting the risk of OC. For this purpose, four tumour-suppressor genes: *Ras Association Domain Family Member 1* (*RASSF1*), *Paired Box 1* (*PAX1*), *Cadherin 1* (*CDH1*) and *Phosphatase and Tensin Homolog* (*PTEN*) were analysed.

*RASSF1* inactivation is connected with the development and progression of many cancer types, and while this inactivation can be caused by deletions and point mutations, it most often arises from methylation changes [14,15]. *RASSF1* encodes a protein similar to the RAS effector proteins [16] and this product regulates cell-cycle progression and apoptosis pathways, especially the Ras/PI3K/AKT, Ras/RAF/MEK/ERK and Hippo apoptosis pathways [16]. It also regulates Bax-mediated cell death [17], interacts with the *XPA* DNA repair gene [18] and inhibits accumulation of cyclin D1 [19]. In addition, it inhibits anaphase-promoting complex (APC) activity and mitotic progression following interaction with CDC20 [20]. *PTEN* is a multifunctional tumour-suppressor gene encoding phosphatidylinositol-3,4,5-trisphosphate 3-phosphatase, which de-phosphorylates phosphoinositide substrates, and it is also an important regulator of insulin signalling, glucose metabolism and the PI3K/AKT/mTOR pathway [21]. The *CDH1* gene encodes the cadherin–calcium dependent cell adhesion protein which regulates the mobility and proliferation of epithelial cells [22], and abnormalities in this gene are connected with the development of colorectal [22], breast [23], gastric [24] and most likely also OC [25]. The *PAX1* gene is a member of the paired-box transcriptional factor gene family which regulates proper tissue development and cellular differentiation in embryos [26]. Although its role in cancer development and progression has not been sufficiently established [27], this gene is aberrantly hyper-methylated and down-regulated in different types of cancer [28], and is suggested to inhibit the phosphorylation of multiple kinases, especially after challenges with the *EGF* and *IL6* oncogenic growth factors [28]. In addition, *PAX1* has the ability to activate variable phosphatases, including DUSP-1, 5, and 6, and to inhibit EGF/MAPK signaling. Its interaction with *SET1B* increases histone H3K4 methylation and the DNA demethylation of numerous phosphatase-encoding genes [28].

Finally, changes in DNA methylation were repeatedly noted in OC and these affected the activity of variable genes, and therefore contributed to the tumorigenesis of variable histotypes. Furthermore, methylation changes in OC can be prognostic for shorter progression-free survival (PFS) and chemotherapy resistance [29,30,31]. Importantly for this study, all four selected genes have previously been observed to be abnormally methylated in primary OC tumours [25,32,33,34,35,36,37,38,39,40]. Moreover, lower mRNA expression in at least one OC histotype in the *RASSF1* and *PTEN* genes [41,42,43] and lower *CDH1* gene protein expression [44] in OC have been noted. Decreased *CDH1* gene expression also correlated with the methylation levels and all these genes can, therefore, be suitable candidates for methylation analysis of LB samples from women with OC.

## 2. Material and Methods

Our sample collection consists of 128 variable types of ovarian tissues and paired plasma samples from central European caucasian women who underwent surgical excision as part of their treatment. This includes healthy (mean age 63.83 ± 11.23 years), benign (64.57 ± 16.40 years) and malignant samples (57.20 ± 11.12 years), and also a few ovarian cancers subsequent to breast cancer (BC-OC) (59.25 ± 12.74 years) (Table 1). The histological characteristics of the OC samples are summarised by FIGO staging and subtype in Table 2 and Table 3. Personal and gynaecological anamnesis information was obtained during medical examination and the study was approved by the ethical committee of Jessenius Faculty of Medicine in Martin Slovakia under number 1933/2016, and the research was performed in compliance with the Declaration of Helsinki.

### 2.1. DNA Isolation and Bisulfite Conversion

Immediately after section, the OT and healthy control tissues were stabilised in RNAlater solution and kept at −20 °C. DNA from tissue and plasma was isolated by DNeasy Blood and Tissue Kit (Qiagen GmbH, Hilden, Germany) and QIAamp^®^ DSP Virus Kit (Qiagen GmbH, Hilden, Germany). The concentration was measured by Nanodrop^TM^ 2000 (Thermo Fisher Inc, Wilmington, DE, USA) and QuBit (Thermo Fisher Inc, Wilmington, DE, USA). Finally, subsequent bisulfite DNA conversion was performed by the Epitect Bisulfite Kit (Qiagen Inc., Valencia, CA, USA) and the converted DNA was stored at −20 °C.

### 2.2. Methylation Analyses

Methylation of bisulfite modified DNA from control tissues, primary tumours and plasma samples were analysed by pyrosequencing on Pyromark Q96ID (Qiagen GmbH, Hilden, Germany). This is a very practical and reliable method and it provides quantitave methylation levels relatively quickly.

Sequencing was preceded by PCR amplification by Pyromark PCR Kit^®^ (Qiagen GmbH). The total reaction volume was 25 µL and comprised 21 µL Pyromark PCR Kit mixture (12.5 µL MasterMix, 2.5 µL CoralLoad Concetrate, 1µL MgCl_2_, 5µL Q-Solution), 2.5 µL primer pairs, 0.5 µL RNAse free water and 1 µL bisulfite modified DNA.

The PCR reaction steps were as follows: Activation of polymerase (95 °C, 15 min), 45 cycles (*RASSF1*) or 50 cycles (*PTEN*, *CDH1*, *PAX1*) of denaturation (94 °C, 15 s), annealing (56 °C (*RASSF1*, *PAX1*)/62 °C (*PTEN*, *CDH1*) for 30 s), extension (72 °C, 30 s) and final extension at 72 °C for 10 min. The qualitative and quantitative amplicon parameters were subsequently assessed by 1.5% agarose gel electrophoresis. The amplification process was the same for both DNA from tumorous tissue and circulating DNA. The PCR amplification primers were modified so that the reverse primer had a biotinylated 5′ end. This enabled separation of the strand for pyrosequencing analysis.

The PCR product was subsequently used for pyrosequencing. This was first mixed with streptavidin-coated sepharose beads (GE Healthcare Life Sciences, Chalfont, UK), binding buffer (Qiagen GmbH) and nuclease free water in a total volume of 80 µL in a Pyromark plate and shaken for 10 min at 2,500 rpm. The 5′-biotinylated strand for sequencing was immobilised by Pyromark working station, transferred to a mixture of 0.4 M sequencing primer and binding buffer (Qiagen GmbH) and incubated for 2 min at 80 °C. Finally, the pyrosequencing results were interpreted by Pyromark Q96 software version 2.5.8 (Qiagen GmbH). The commercially available CpG methylation assays^®^ (Qiagen GmbH) were used herein to analyse methylation levels in the following sequences (Table 4):

### 2.3. Biostatistical Analyses

Exploratory data analysis was performed by standard summary statistics; the methylation data was visualised by boxplot overlain with swarmplot; and the heat map provided methylation level mean values. Data normality was assessed by a quantile-quantile plot with 95% bootstrap confidence band, and robust ANOVA tested the hypothesis of equality of population methylation medians in the disease levels. Null hypothesis rejection was followed by the Tukey HSD post-hoc test. The discriminative ability of the methylation indices and age for various pairs of disease level was then assessed by the random forest learning algorithm. This quantified the importance of the individual predictors by minimal depth. The trained random forest discriminative ability was visualised by the ROC curve based on “out-of-bag” data and the AUC area under the ROC curve and the 95% AUC confidence interval quantified the discriminative ability of methylation indices and age. Finally, we determined sample size and performed power analysis for the one-way ANOVA test, assuming balanced design. All data analysis was performed in R, version 3.5.2 [45].

## 3. Results

### 3.1. CpG Sites Methylation Status of the RASSF1, PTEN, CDH1 and PAX1 Gene Regulatory Regions

DNA methylation profiling of 21 CpG sites in four genes, comprising six CpG sites in the *RASSF1* gene and five CpG sites in each of the *PTEN*, *CDH1* and *PAX1* genes was performed by pyrosequencing on the four different tissue types—healthy ovarian tissues, benign OT, OC and BC-OC, and also on the corresponding plasma samples. The mean value, median and standard deviation of each CpG site is provided in Supplementary Table S1 for tissues and Table S2 for plasma.

**Figure 1:**
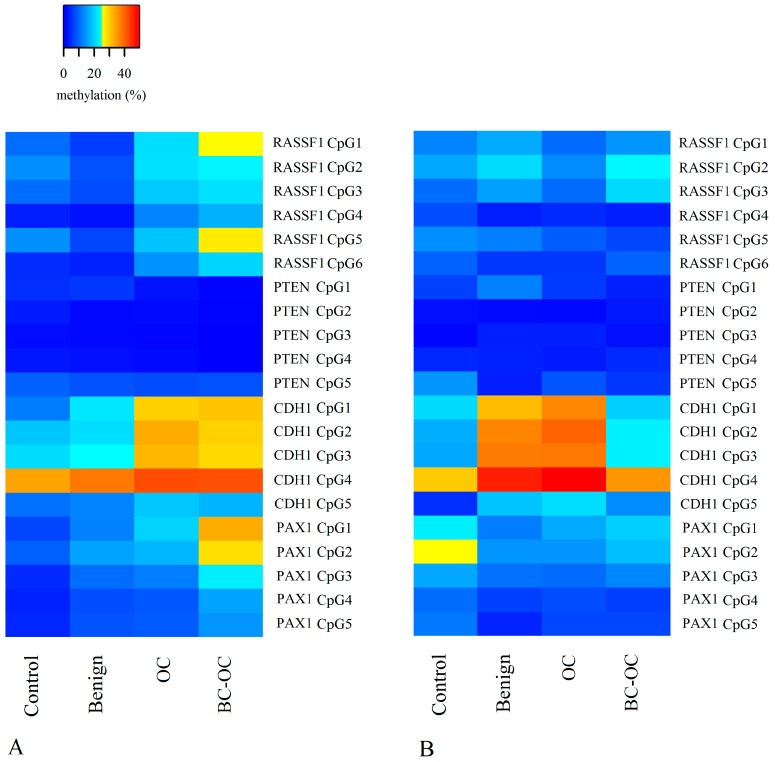
Heat map of each CpG site methylation values in tissue (**A**) and plasma samples (**B**) according to diagnosis. OC—ovarian cancer; BC-OC—ovarian cancer subsequent to breast cancer.

Figure 1 shows the methylation value of each CpG site in the tissue (A) and plasma samples (B) according to diagnosis. The general overview provides information on the hypomethylation of *PTEN* CpG sites for all diagnoses and tissue and plasma samples. Greater variability is obvious in the *RASSF1* and *PAX1* CpG sites, and the highest methylation was detected in the *CDH1* CpG sites. The comparison of tissue samples (Figure 1A) shows the increasing methylation trend from the control sample group to cancer samples in the *RASSF1*, *CDH1* and *PAX1* genes. The statistical comparison of means revealed the most significant differences between controls and OC samples in two *CDH1* and three *PAX1* gene CpG sites and between controls and BC-OC in three *PAX1* gene CpG sites. The increasing mean values for *RASSF1* were not statistically significant because of the wide variance in values (detailed statistical data is contained in Table S1). Figure 1B depicts variability in the plasma group methylation values, with no visible linear trends detected between the diagnostic groups. Finally, the statistically significant higher methylation levels were detected in OCs in four *CDH1* gene CpG sites compared to controls, and all detailed statistical data is listed in Table S2.

### 3.2. DNA Methylation Indices of the RASSF1, PTEN, CDH1 and PAX1 Gene Regulatory Regions

**Figure 2:**
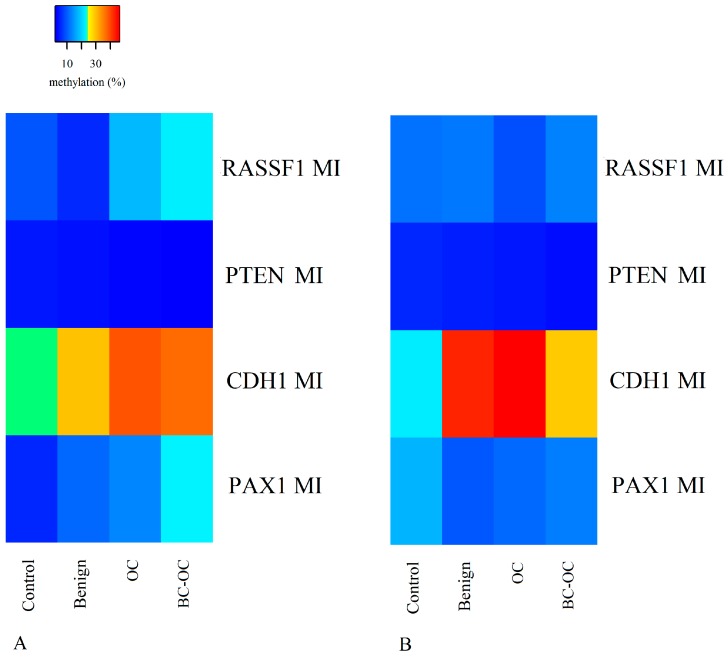
Heat map of MI values in tissue (**A**) and plasma (**B**) samples according to diagnosis. MI—methylation index, OC—ovarian cancer; BC-OC—ovarian cancer subsequent to breast cancer.

The methylation index (MI) is the mean percentage of methylation across all CpG sites analysed per gene, and that was calculated for each gene and diagnostic group in the tissue and plasma samples. The MI values of each diagnostic group are depicted by the heat maps in Figure 2A for tissue and 2B for plasma. Although *PTEN* gene methylation indices were very low, they were statistically significantly different between benign tumours (3.90 ± 4.10) and malignant OC (3.00 ± 2.60) in the tissue samples. No statistically significant differences were determined in *RASSF1* MI between diagnostic groups in either the tissue or plasma samples. In the tissue samples, the *CDH1* and *PAX1* gene methylation indices had increasing tendency toward OC, with statistically significant difference between controls (24.22 ± 6.93 for *CDH1* and 5.73 ± 2.19 for *PAX1*) and OCs (39.06 ± 18.78 and 13.55 ± 10.65) in both genes, and also between controls (5.73 ± 2.19) and BC-OC samples (22.90 ± 13.56) in the *PAX1* gene. In the plasma samples, there was only one statistically significant difference between controls (22.25 ± 14.13) and OC samples (46.42 ± 20.91), in the *CDH1* gene. Detailed data is provided in Supplementary Table S3.

**Figure 3:**
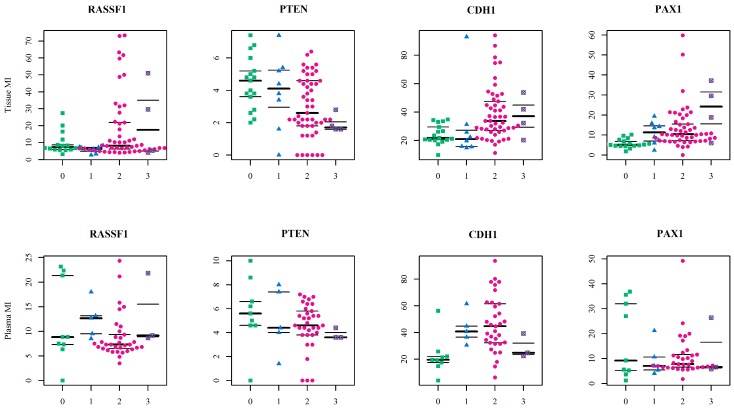
The swarmplots of methylation indices of *RASSF1*, *PTEN*, *CDH1* and *PAX1* genes, showing the mean values of all observations. MI—methylation index; 0—control tissues (green squares); 1—benign tumours (blue triangles); 2—ovarian cancers (pink circles); 3—ovarian cancers subsequent to breast cancer (purple crossed circles).

Analysed sample distribution is depicted in Figure 3 according to the sample type and diagnosis for all four studied genes. Wider variance in the mean values is obvious in the *PTEN* methylation index, and that explains the small differences between groups.

### 3.3. Methylation Pattern by FIGO Staging and Histological Type

The methylation levels of all CpG sites revealed little statistically significant differences between the compared FIGO stages (data not shown) and the MI data pattern is depicted in Table 5. Two following trends were observed: The *RASSF1* and *PAX1* methylation indices decreased with disease severity increase, and the *CDH1* and *PTEN* values had increasing tendency, thus followiing the stages. Statistical comparison of the mean values revealed significant differences only in the *PTEN* gene and between stages I and II, and stages I and IV in the *PTEN* gene.

Comparison of the methylation levels and indices between histological subtypes revealed only sporadic differences and indicated possible uniform methylation of the studied genes in this OC group.

### 3.4. Comparison of Methylation Indices in Tissue and Plasma Paired Samples

**Figure 4:**
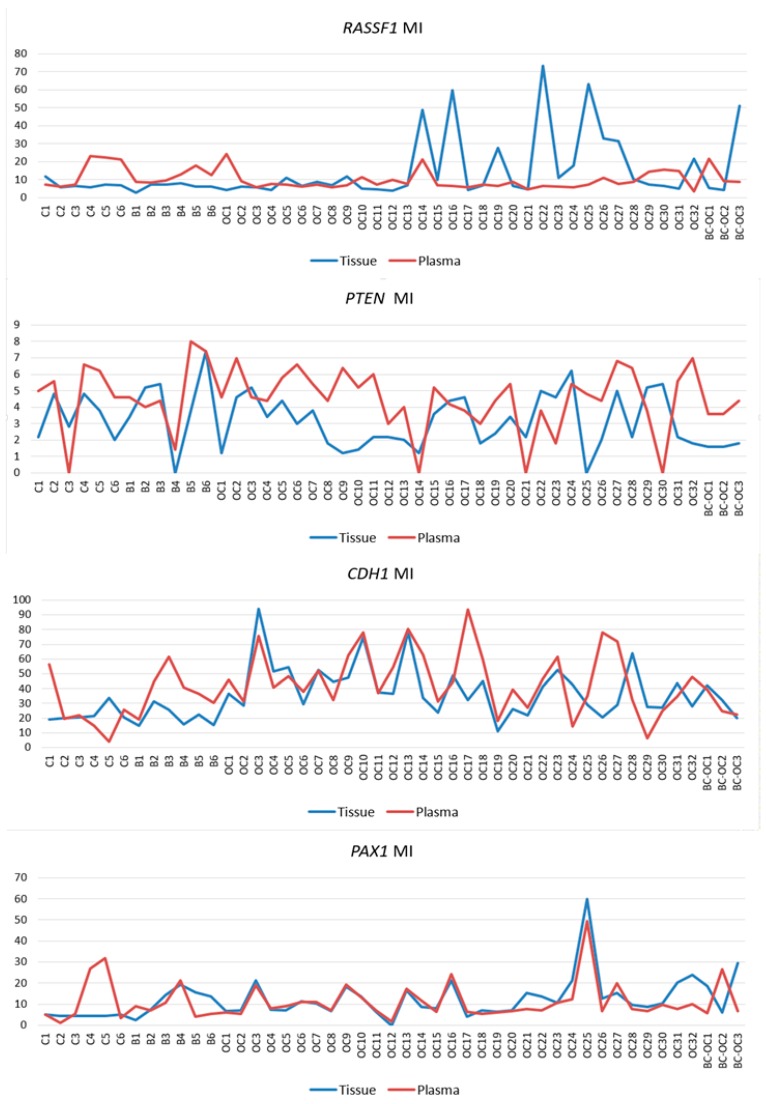
Methylation indices of *RASSF1*, *PTEN*, *CDH1* and *PAX1* genes in paired tissue and plasma samples. MI—methylation index; C—control samples; B—benign; OC—ovarian cancer; BC-OC—ovarian cancer subsequent to breast cancer.

The MI values of paired tissue and plasma samples were compared to assess if the plasma methylation status reflects those observed in the primary tumours. Figure 4 shows similarities and differences in the methylation indices of all *RASSF1*, *PTEN*, *CDH1* and *PAX1* gene paired samples, and statistical analysis determined significant plasma and tissue correlation only in the *CDH1* and *PAX1* genes. The correlation coefficient of *CDH1* MI was negative in controls (*r* = −0.812, *p* = 0.05), but positive in benign samples (*r* = 0.886, *p* = 0.019) and OC samples (*r* = 0.428, *p* = 0.015). However, strong correlation was established between plasma and tissue in all *PAX1* CpG sites and between *PAX1* methylation indices, but only in the OC pairs (*r* = 0.771, *p* < 0.001).

The results for sample size determination, including the effect size and the number of observations in each group needed to maintain 80% power level, are included in Supplementary Table S4 for tissue samples and Table S5 for plasma samples.

### 3.5. Evaluation of the Diagnostic Predictive Model Using ROC Analyses

Finally, we used the random forest algorithm with ROC analysis to evaluate if the studied methylation indices combined with age could significantly distinguish between diagnoses. Table 6 provides the AUC values with 95% confidence intervals for tandem discriminations. The “Important variable” column denotes the most relevant variables based on importance measured by minimal graph depth. The best AUC values were obtained in the model which discriminate between controls and OC samples based on both the MI of the four genes in tissues (AUC = 0.932) and in plasma (AUC = 0.822). The first “tissue” model had perfect sensitivity (98%), but specificity was weak (34%). The same was observed in the “plasma” model, with 91% sensitivity and 56% specificity. Based on the AUC values and 95% CI, these models indicate strong predictive power, and can, therefore, be considered for diagnostic discrimination clinically.

## 4. Discussion

OC is one of the most lethal cancerous diseases, and despite the progress in surgical techniques, better management, advanced chemotherapy and targeted therapy, the OC patient survival rate is still very low and almost 70% of all patients are diagnosed in advanced stages. This is mainly due to the lack of early symptoms in this disease [8]. Early OC screening approaches have been traditionally based on serum CA-125 concentration and trans-vaginal ultrasound, but both these methods lack sufficient sensitivity and specificity [46,47]. Therefore, non-invasive biomarkers able to predict early OC presence and monitor response to treatment and cancer progression are imperative. However, those with sufficient predictive power are still lacking and most possible candidates require further investigation [46,48,49]. Moreover, further use of the tumour markers is crucial in all gynaecological cancerous diseases [50,51,52]. For example, CTC, ctDNA, cfmiRNAs and released exosome vesicles can be analysed in the assessment of molecular markers. This assessment can be performed by a variety of methods, from basic PCR modifications to advanced multi-parallel sequencing approaches [10].

Herein, we focused on the epigenetic impact on OC formation by analysing the methylation levels of the *RASSF1*, *PTEN*, *CDH1* and *PAX1* tumour suppressor genes’ regulatory sequences. We compared the methylation status of normal tissue, benign, OC and BC-OC samples. We then assessed the methylation levels in these genes in the corresponding plasma samples in order to estimate their potential for LB utilisation. Finally, we compared the methylation patterns in the tissue samples and the paired plasma samples to assess if these patterns were markedly different.

Previously published articles reported the importance of some genes’ plasma methylation status in OC management. For example, the *C2 CD4 D*, *WNT6* and *COL23 A1* genes’ methylation status is not only significantly altered in OC patients’ ctDNA, but different responses to platinum-based therapy can also be associated with their methylation levels [53]. Moreover, Giannopolou et al. [54] discovered altered *ESR1* gene methylation levels in the ctDNA samples of high-grade serous OC patients, and these abnormalities are very similar to those in primary tumours [54].

The most notable methylation changes in solid OT are in the *BRCA1* [29,55], *OPCML* [56], *HOXA9* [33] and *P16 INK4α* genes [57]. *BRCA1* is methylated in up to 30% [29,55] of these tumours and *OPCML* is methylated in over 80% of OT [56]. Interestingly, *RASSF1 A* is the major *RASSF1* gene isoform and this has been repeatedly analysed for methylation changes in both OC tissues and plasma samples. For example, de Caceres et al. [32] noted methylation of the *RASSF1 A* gene in 50% of primary OT and detected an identical pattern of gene hyper-methylation in 41 of 50 (82%) matched plasma DNA. In addition, hyper-methylation of this gene was recorded in all OC histological types, grades and stages. Wu et al. [33] reported very similar results in solid tissues, with hyper-methylation in 49% of patients with various OC types and grades. In contrast, Agathanggelou et al. [58] found hyper-methylation in only 10% of solid OT.

Methylation changes in the *RASSF1 A* promoter have already been analysed for utilization in non-invasive differentiation of epithelial ovarian carcinoma from healthy controls. For example, 90% sensitivity and 86% specificity for cancer detection was determined when methylation levels of the *RASSF1 A*, *EP300* and *CALCA* genes were used in combination [59]. In addition, the multiplex methylation specific PCR (MSP) assay of seven different genes, including *RASSF1 A*, achieved 85.3% sensitivity and 90.5% specificity in differentiating epithelial OC from healthy controls in serum samples [60]. Variable *RASSF1 A* methylation has also been noted in tissue samples of serous and non-serous types and benign tumours [61].

Herein, the *RASSF1 A* MI differed in all four tissue types. It was higher in primary malignant tumours (17.98 ± 19.73) than in healthy tissue (9.50 ± 6.21), but the opposite was found in plasma ctDNA. Here, the controls have higher MI (11.74 ± 8.34) compared to malignant samples (8.93 ± 4.54). However, no statistically significant differences were established between particular CpGs or mean methylation indices. Importantly, although these differences proved insignificant, they can indicate wide methylation changes in *RASSF1* gene regulating sequences and the important role of methylation in silencing this gene in OC development as is generally suggested [29].

Although the methylation status of the *PTEN* CpG sites and methylation indices was generally low and homogenous, there were statistically significant differences between benign and malignant lesions. The decreasing tendency in methylation indices was observed in both tissue and plasma samples, but comparison between FIGO stages revealed a statistically significant increase in methylation indices only in the higher FIGO stages. This theoretically indicates its important role in cancer formation and spread. Herein, however, we compared only the main FIGO categories where subtypes were merged in one main category, because of the small sample size. In addition, the differences between the FIGO stages were not only low, but the methylation indices themselves also had low values. This leads to debate if such small increases in methylation levels can contribute to tumour progression from stage I to stage IV. This is especially pertinent when previous very low *PTEN* methylation levels have occurred without differences in OC FIGO stages [34].

Yang et al. [34] and Zuberi et al. [35] recorded relatively low methylation values in the *PTEN* gene with higher methylation in only 16% and 8.2% of the samples, respectively. Schondorf et al. [62] suggested that *PTEN* methylation should have only a subordinate role in OC progression and only the one study by Qi et al. [36] reported relatively higher methylation levels of this gene in OC. Overall, the low and homogeneous *PTEN* methylation concord with the most of previous studies, and the small increase in methylation should not reflect progression from stage I to stage IV, though statistical significance is established between the FIGO stages. Our results also indicate the potential effect of *PTEN* methylation changes in benign lesion formation. That suggests that further analysis of the methylation changes will be beneficial, because there is no rule that benign lesions must be less methylated than malignant lesions, and different mechanisms can lead to abnormal methylation in benign and malignant tumours [63].

In comparison to *RASSF1* and *PTEN*, the *PAX1* gene had greater variation in methylation levels with statistically significant differences between controls, OC and BC-OC. There was also wide variability in particular CpG islands, especially in the control and malignant samples. This gene’s methylation provides a potential biomarker for cervical cancer screening [64,65,66], but important methylation changes in this gene were also noted in OC. In particular, Su et al. [37] reported 50% *PAX1* methylation rates in OC, but only 14.3% in borderline tumours and 4% in benign, and Hassan et al. [38] found hyper-methylation in 50%, 50%, 46.6% and 78% of patients depending on the FIGO stage of epithelial OC. In addition, the authors associated methylation levels with the presence of HPV16/18 infection. Neither of these studies can be quantified in the same manner as our quantitative pyrosequencing results because they used MSP in their assessment, but the significant differences we achieved between healthy tissues and cancer samples can be interpreted quite similarly—that dynamic methylation changes indicating OC’s presence occur in the aforementioned gene’s regulation sequences. Although our results established no statistical significance, they can certainly help move the debate on how this gene’s methylation changes affect the formation of benign lesion because the methylation levels were as high they are in cancerous tissues.

Finally, the *CDH1* gene had statistically significant variability in methylation levels between control and malignant tissue samples, and this agrees with previous analyses [25,39,40]. Importantly, this gene also had statistically significant plasma samples differences in both mean methylation levels and those of particular CpG dinucleotides in both healthy and cancerous groups. Therefore, the *CDH1* gene could be considered a very promising candidate for non-invasive LB-based diagnosis of malignant OC, but its potential for non-invasive investigation of benign tumour presence requires further analysis.

It is important to realize that even non-significant, but observable differences in plasma samples methylation levels were revealed also in the *RASSF1*, *PTEN* and *PAX1* genes, and this could inspire additional analyses of these genes. The large deviation in the final methylation levels of these genes was very likely the main obstacle to achieving statistical significance. However, larger sample collection could help to solve this problem.

One of the issues in the analysis of plasma samples is the lack of definition and accepted consensus if changes, including those in ctDNA methylation, remain exactly the same as in the primary tumours that released them. While some authors consider that the aberrant patterns remain identical to those in the primary tumour [53,67,68,69,70,71,72], others support diverse patterns [73,74,75]. Herein, we conform to similarity because of the strong correlations between tissue and plasma samples in the *CDH1* and *PAX1* genes, and also in several *RASSF1* and *PTEN* gene samples. Discrepancies in tissue and plasma sample methylation levels could have also occurred herein because circulating cfDNA of cancer patients contains a mixture of DNA from both normal and cancerous cells. The ratio of ctDNA in the entire cfDNA fraction can vary because this depends on several tumour parameters [76]. Our methodology cannot estimate how plasma sample results are affected by the presence of non-tumorous cfDNA elements, and we cannot exclude that this is responsible for the variability obtained in the results. Although the ctDNA ratio is generally quite low it can vary widely, and some estimations of ctDNA abundance reach 90% [76,77]. Importantly, it has been demonstrated that the concentration of all circulating free molecules, and not only ctDNA, is associated with a tumour volume which gives shorter overall survival (OS) for OC patients [78].

Previously performed LB-based methylation analyses assessing the methylation pattern in OCs using non-NGS approaches have provided very interesting results. Those include (1) Teschendorff et al. [79], when they used a methylation-array based study and observed significant differences in the blood of healthy and cancerous patients, and (2) Flanagan et al. [80], who utilised the pyrosequencing approach and determined significant correlation of *SFN* gene methylation with PFS. In addition, their subsequent study [81] noted specific CpGs’ alterations in blood DNA following relapse from platinum-based chemotherapy, and their results proved an independent significant association with survival. Moreover, both pyrosequencing and MSP have previously been used to assess methylation abnormalities in plasma samples from different cancer types, and some of these achieved significant results assessing the profiles of all circulating DNAs, not only ctDNA [82,83,84,85,86]. Therefore, even methodology which is unable to distinguish ctDNAs in the whole cfDNA fraction can be useful in assessing disease characteristics from LB samples. We also incline toward the consensus that changes in primary tumours should remain preserved in ctDNA elements and the changes noted in primary tumours could then be used as inspiration for initiating further experiments utilising LB samples. Therefore, known abnormalities in primary tumours can be very helpful because these can be searched and analysed in LB samples.

OC is widely recognised as a very heterogeneous group and it should, therefore, be important to analyse and interpret the results of each histotype. However, the rarity of some morphological subtypes causes study limitations, and the small numbers in some histotypes herein prevented performance of statistical analysis. Only the serous and serous papillary histotypes were sufficient for analysis, and these recorded no significant differences in their methylation levels and were even almost identical. While studies have confirmed that some differences are typical for all OC histotypes [25,32,33,34,56], it cannot be ignored that unintentional variance in our results is due to the heterogeneity of our collection and also the impossibility of separating and analysing particular histotypes.

The effect size and the number of observations in each group required to maintain 80% power were assessed by power analysis, and these are listed in supplementary Tables S4 and S5. Herein, the number of malignant tissues was mostly sufficient, but we occasionally noted a lack in the number of healthy controls and the number of benign lesions was quite often lower than proposed. Therefore, although the power analysis results may limit our study through having a sample size insufficient to assess methylation levels in all CpGs with sufficient statistical power, it can help design the same or similar experiments. The highest number of required observations, excluding three *PTEN* gene CpG sites in plasma samples, was 64, and it is quite possible to collect at least that number of samples for future analysis.

It has been noted that there are generally higher cfDNA levels in malignant OC patients than in healthy patients and in those with benign lesions [87,88]. The cfDNA levels are also lower in FIGO I–II stages than in the III–IV stages [78]. Although we assessed only four genes’ methylation indices and not the entire cfDNA levels, and therefore cannot directly associate these parameters, analysis of the relationship between methylation changes, tumour stage and general cfDNA levels could most likely provide clinical implications. It would be beneficial, therefore, to design further studies which assess these relationships.

The cfDNA characteristics can also vary depending on age [89] and the presence of concomitant disease [90]. Furthermore, it is presumed that length of cfDNA fragments correlates with shorter PFS, and also OS [91]. Unfortunately, we can neither confirm nor preclude that our results are affected by other clinical attributes, such as the presence or absence of other patient disease. However, further studies should establish associations between a wide variety of clinical parameters, methylation and also cfDNA levels, as previously suggested.

Herein we evaluated the predictive performance of the combined model of DNA methylation signature (MI of the genes) and the age (in years) using ROC analyses computed by the random forest algorithm. We ran six models separated for sample type. The model which discriminated between control and OC samples had the highest predictive power—at 0.932 AUC in tissue samples and 0.822 in plasma. This initially appeared a perfect model with high sensitivity and clinical application potential, but 34% specificity for tissue samples and 56% for plasma samples means that too many healthy women would be incorrectly classified as cancer patients. The most likely reason for these results was the small number of controls, and this could have affected statistical analyses and hindered efforts to ensure a strong statistical model. Adequate AUC values were obtained in the model discriminating control samples and benign samples (tissue: AUC = 0.729, 85.7% sensitivity and 83.3% specificity; plasma: AUC = 0.778, 100% sensitivity and 66.7% specificity). Our last discrimination model of benign and OC samples had insufficient classifiers. In these models, *PAX1* MI and *CDH1* MI were selected as the most important variables for tissue and plasma respectively, and this indicates the previously described results about tissue and plasma non-conformity. Although these analyses promised a high-quality classifier, other possibilities should now be also considered. The most important would be the inclusion of additional control samples, as this would provide more accurate specificity. It is also worth considering the inclusion of more variables in the model, such as gynaecological anamnesis, life style risk factors and also CA-125 or other novel biomarkers with the potential to detect serous OC at earlier stages and predict patient survival prognosis [92,93,94]. Although this is beyond the scope of this pilot project, it should provide inspiration for further analyses focused on specific variables identified by a multinomial approach and offer personalised tailored therapy. Because of the great variability in this disease, the management of OC should be individualized, and performance status of the patient should be considered. This is best reflected in necessity for different treatment option for younger and older women [95,96]. In addition, future individual OC treatment should also be based on different patient physiological and health parameters.

In conclusion, our study confirmed higher methylation levels in several CpG sites and also higher methylation index in *RASSF1 A*, *PAX1* and *CDH1* genes in OC tissues compared to control tissues. However, no statistical significance was noted in the *RASSF1* gene because of high variance. Statistically significant higher MI and CpG sites methylation levels were detected in plasma samples only in the *CDH1* gene. *PTEN* gene was slightly and homogenously methylated. In addition, we recorded similar methylation patterns with strong correlations in the *CDH1* and *PAX1* genes in OC plasma and tissue, and the results suggest that the *CDH1* gene is a prospective candidate for non-invasive, LB-based differentiation in OC. Our diagnostic discrimination model combined the methylation indices of all four genes with age, and this revealed the highest AUC 0.932 predictive power in the model, comparing OC subjects with controls. Finally, it will prove highly beneficial to employ as many LB-based approaches as possible in cancer detection and treatment, especially in OC, which has such high risk and generally delayed diagnosis. LB is capable of providing early OC diagnosis which can help decrease the global expense of fighting this disease, but most importantly it can save lives, or at least prolong them with greater life quality for the thousands of women affected by this lethal cancer.

## Figures and Tables

**Table 1:** Number of samples according to the diagnosis.

Tissue		Diagnosis	Plasma		Paired Samples
*n*	%		*n*	%	*n*
17	13.3	Control	9	7.0	7
8	6.3	Benign	5	3.9	5
49	38.3	OC	33	25.8	33
4	3.1	BC-OC	3	2.3	3

Note: OC—ovarian cancer; BC-OC—ovarian cancer subsequent to breast cancer.

**Table 2:** FIGO classification of OC samples.

Stage	*n*	%
1A	5	10.2
1C	3	6.1
1C2	1	2.0
3b	1	2.0
3B	3	6.1
3C	17	34.7
4	7	14.3
4B	5	10.2
No data	7	14.3

**Table 3:** Histopathological subtypes of OC samples.

Subtype	*n*	%
Clear cell	2	4.1
Endometrioid	2	4.1
Mucinous	4	8.2
Serous	16	32.7
Serous papillary	25	51.0

**Table 4:** Analysed sequences of genes *RASSF1*, *PTEN*, *CDH1* and *PAX*, and their location in human genome.

Gene	Sequence to Analyse	Location
***RASSF1***	5′-GGTAGCGCAGTCGCCGCGGGTCAAGTCGCGGC-3′	3:50,377,907–50,377,938
***PTEN***	5′-CGCGAGGCGAGGATAACGAGCTAAGCCTCGGC-3′	10:89,622,923–89,622,954
***CDH1***	5′-CGGCAGCGCGCCCTCACCTCTGCCCAGGACGCGGC-3′	16:68,772,300–68,772,331
***PAX1***	5′-CGGAATCTGCTAGCTTCGTCGGGCGCGA-3′	20:21,684,296–21,684,323

**Table 5:** MI averages of CpG sites in regulatory regions of the *RASSF1*, *PTEN*, *CDH1* and *PAX1* genes in tissue samples according to FIGO stage.

	Stage I (*n* = 9)			Stage II (*n* = 21)			Stage IV (*n* = 12)			*p*-Value
	Mean	Median	SD	Mean	Median	SD	Mean	Median	SD	
**MI_*RASSF1***	19.24	5.33	20.46	17.24	7.50	19.80	8.01	6.83	3.95	ns
**MI_*PTEN***	2.09	2.00	1.37	3.47	3.60	1.49	3.63	3.60	1.37	a, b
**MI_*CDH1***	32.89	29.00	16.81	39.98	30.50	20.72	48.38	44.25	17.76	ns
**MI_*PAX1***	17.11	13.60	17.36	11.56	10.40	5.42	10.98	8.50	6.28	ns

Note: Due to number of cases in each category, we created only three groups, based on the FIGO stage numbers (for example 1A, 1B and 1C were merged into stage I); ns—non significant difference between groups; a—statistically significant difference (*p* < 0.05) between FIGO I and II; b—between FIGO I and IV.

**Table 6:** Predictive performance of the model distinguishing diagnostic groups.

		AUC	95% CI	Overall Error Rate (%)	Important Variable
Tissue	Control vs. Benign	0.738	0.391–1.000	15.38	*PAX1*
	Control vs. OC	0.932	0.823–1.000	9.09	*PAX1*
	Benign vs. OC	0.499	0.195–0.802	12.5	*CDH1*
Plasma	Control vs. Benign	0.778	0.512–1.000	21.43	*CDH1*
	Control vs. OC	0.822	0.667–0.976	16.67	*CDH1*
	Benign vs. OC	0.630	0.309–0.951	13.16	*PTEN*

Note: OC—ovarian cancer, AUC—area under the curve, CI—confidence interval.

## Supplementary Materials

Supplementary Materials can be found at https://www.mdpi.com/1422-0067/20/17/4119/s1.

## Abbreviations

AUC	Area under the curve
BC-OC	Ovarian cancer subsequent to breast cancer
CDH1	Cadherin 1
cfmiRNA	Circulating cell-free microRNAs
CTC	Circulating tumour cells
ctDNA	Circulating tumour DNA
LB	Liquid biopsy
MI	Methylation index
MSP	Methylation specific PCR
OC	Ovarian cancer
OS	Overall survival
OT	Ovarian tumours
PAX1	Paired Box 1
PFS	Progression-free survival
PTEN	Phosphatase and Tensin Homolog
RASSF1	Ras Association Domain Family Member 1
ROC	Receiver operating characteristic
