# Supplementary material for: Aberrant Methylation Status of Tumour Suppressor Genes in Ovarian Cancer Tissue and Paired Plasma Samples
Source: Int J Mol Sci. 2019 Aug 23;20(17):4119. doi: 10.3390/ijms20174119 (PMC6747242; doi:10.3390/ijms20174119)
Supplement: Supplementary file 1 [file ijms-20-04119-s001.pdf]

Table S1. DNA methylation level of CpG sites in promoter regions of genes *RASSF1*, *PTEN1*, *CDH1* and *PAX1* in tissue samples

Gene	Control			Benign			OC			BC-OC			P-value
	**Mean**	**Median**	**SD**	**Mean**	**Median**	**SD**	**Mean**	**Median**	**SD**	**Mean**	**Median**	**SD**	
RASSF1													
CpG1	10.65	9.00	11.89	6.38	7.50	2.62	21.24	11.00	23.40	25.50	18.00	27.05	ns
CpG2	13.71	9.00	14.56	8.00	7.50	2.14	21.59	11.00	21.09	23.75	19.50	21.23	ns
CpG3	10.53	9.00	11.19	7.50	8.50	2.00	19.41	10.00	20.43	21.50	18.00	19.47	ns
CpG4	3.41	3.00	3.06	2.50	3.00	1.60	12.78	4.00	18.83	17.00	15.00	16.69	ns
CpG5	13.88	8.00	18.00	6.88	7.00	2.53	18.71	9.00	21.28	27.00	21.00	26.85	ns
CpG6	4.82	4.00	5.03	3.75	4.00	2.19	14.16	6.00	16.87	20.50	14.00	23.23	ns
PTEN1													
CpG1	4.88	5.00	1.90	5.75	3.50	7.96	2.67	3.00	2.05	1.00	1.00	1.15	d
CpG2	3.12	3.00	1.83	1.50	1.50	1.60	1.86	2.00	1.80	0.75	0.00	1.50	ns
CpG3	2.12	3.00	2.03	1.75	2.00	1.58	1.33	0.00	2.07	0.00	0.00	0.00	ns
CpG4	2.88	4.00	2.34	2.25	2.50	1.67	1.76	0.00	2.46	0.00	0.00	0.00	ns
CpG5	9.35	10.00	3.53	8.25	9.00	3.88	7.41	8.00	3.86	8.00	6.00	4.00	ns
CDH1													
CpG1	11.94	11.00	4.79	22.38	13.50	21.02	29.67	26.00	16.85	30.75	33.50	11.87	b
CpG2	19.06	17.00	8.83	21.38	12.50	26.25	33.12	27.00	17.52	29.50	28.00	13.99	b
CpG3	21.18	19.00	8.43	24.38	16.50	24.86	31.96	26.00	20.09	28.75	28.00	13.33	ns
CpG4	33.94	37.00	7.90	38.13	33.00	19.44	42.24	41.00	14.29	41.75	41.50	12.31	ns
CpG5	10.76	11.00	4.04	12.75	8.50	16.14	19.24	15.00	11.72	17.50	17.50	7.59	b
PAX1													
CpG1	6.88	6.00	3.43	12.50	13.50	6.99	20.31	18.00	13.56	33.25	29.50	23.27	b, c, e
CpG2	9.24	8.00	3.46	15.63	14.50	8.03	17.65	13.00	13.16	28.25	31.00	16.19	b,c
CpG3	4.59	4.00	1.77	10.63	10.00	6.16	12.27	8.00	10.93	23.00	22.50	15.56	b,c
CpG4	3.82	4.00	2.07	7.75	8.00	5.42	8.73	6.00	8.93	15.75	17.50	9.54	c
CpG5	4.12	4.00	2.09	8.38	8.00	5.95	8.78	6.00	9.39	14.25	15.00	8.18	ns

Note: OC – ovarian cancer; BC-OC – ovarian cancer subsequent to breast cancer; P-value – statistical significance: ns – non significant difference between groups, single letters represent statistical significant difference ($p<0.05$) between groups: a – control and benign, b – control and OC, c – control and BC-OC, d – benign and OC, e – benign and BC-OC, f – OC and BC-OC.

Table S2. DNA methylation level of CpG sites in promoter regions of genes *RASSF1*, *PTEN1*, *CDH1* and *PAX1* in plasma samples												
Gene	**Control**			**Benign**			**OC**			**BC-OC**			**P-value**
	**Mean**	**Median**	**SD**	**Mean**	**Median**	**SD**	**Mean**	**Median**	**SD**	**Mean**	**Median**	**SD**	
RASSF1													
CpG1	12.78	10.00	10.60	16.20	17.00	6.53	10.48	9.00	5.69	14.33	11.00	7.57	ns
CpG2	16.00	11.00	13.32	21.20	18.00	9.63	13.27	10.00	9.09	24.00	13.00	19.92	ns
CpG3	10.67	10.00	7.62	15.20	12.00	12.91	10.52	9.00	5.39	21.00	11.00	19.08	ns
CpG4	7.67	4.00	7.70	3.40	4.00	3.71	4.61	4.00	3.64	3.33	4.00	3.06	ns
CpG5	13.67	10.00	11.48	12.40	10.00	5.94	8.97	8.00	4.50	7.00	10.00	6.08	ns
CpG6	9.67	5.00	8.70	5.80	7.00	3.35	5.73	5.00	3.64	9.67	6.00	7.23	ns
PTEN1													
CpG1	6.56	6.00	4.61	12.60	7.00	9.66	6.00	5.00	7.07	3.67	4.00	0.58	ns
CpG2	2.22	0.00	2.91	1.80	0.00	2.68	1.61	0.00	3.43	3.00	4.00	2.65	ns
CpG3	1.22	0.00	2.54	3.60	0.00	6.50	3.52	0.00	6.82	2.33	3.00	2.08	ns
CpG4	4.11	4.00	4.94	3.80	3.00	4.27	3.21	0.00	4.98	4.67	5.00	1.53	ns
CpG5	14.33	15.00	9.00	3.40	0.00	4.67	8.45	10.00	7.24	5.67	7.00	4.16	d
CDH1													
CpG1	21.00	16.00	14.97	31.60	30.00	13.67	36.55	34.00	19.14	20.00	20.00	6.00	ns
CpG2	16.89	15.00	15.33	37.00	33.00	11.25	39.94	37.00	19.70	23.33	21.00	9.71	b
CpG3	16.11	17.00	15.51	37.80	35.00	9.55	38.15	35.00	20.88	23.33	18.00	9.24	b
CpG4	30.11	30.00	16.74	46.00	43.00	10.32	49.67	48.00	18.20	35.33	34.00	7.09	b
CpG5	4.89	0.00	7.62	18.80	18.00	4.32	21.39	20.00	13.09	13.33	12.00	6.11	b
PAX1													
CpG1	23.11	12.00	22.79	12.20	8.00	11.84	16.18	13.00	11.49	20.00	13.00	16.64	ns
CpG2	25.22	14.00	20.65	14.40	11.00	11.26	14.24	12.00	10.28	18.33	10.00	14.43	ns
CpG3	16.11	9.00	13.67	11.00	7.00	7.38	10.45	8.00	8.31	13.00	6.00	13.00	ns
CpG4	10.67	6.00	8.57	6.60	5.00	4.22	7.45	6.00	6.47	6.33	5.00	7.09	ns
CpG5	11.67	5.00	10.82	4.00	5.00	2.35	6.91	5.00	7.35	7.00	5.00	8.19	ns

Note :OC – ovarian cancer; BC-OC – ovarian cancer subsequent to breast cancer; P-value – statistical significance: ns – non significant difference between groups, single letters represent statistical significant difference (p<0.05) between groups: a – control and benign, b – control and OC, c – control and BC-OC, d – benign and OC, e – benign and BC-OC, f – OC and BC- OC.

Table S3. Methylation index (MI) averages of CpG sites in promoter regions of genes *RASSF1*, *PTEN1*, *CDH1* and *PAX1* in tissue and plasma samples

	Gene	Controls			Benign			OC			BC-OC			P-value
		**Mean**	**Median**	**SD**	**Mean**	**Median**	**SD**	**Mean**	**Median**	**SD**	**Mean**	**Median**	**SD**	
**Tissue**	MI_*RASSF1*	9.50	7.33	6.21	5.83	6.33	1.95	17.98	8.17	19.73	22.54	17.50	22.32	ns
	MI_*PTEN1*	4.47	4.60	1.61	3.90	4.10	2.31	3.00	2.60	1.83	1.95	1.70	0.57	d
	MI_*CDH1*	24.22	21.75	6.93	29.75	21.00	26.18	39.06	33.75	18.78	37.06	37.13	14.24	b
	MI_*PAX1*	5.73	5.20	2.19	10.98	11.30	5.69	13.55	10.40	10.65	22.90	24.20	13.56	b,c
**Plasma**	MI_*RASSF1*	11.74	8.83	8.34	12.37	12.67	3.73	8.93	7.33	4.54	13.22	9.17	7.46	ns
	MI_*PTEN1*	5.69	5.60	2.81	5.04	4.40	2.70	4.56	4.60	1.93	3.87	3.60	0.46	ns
	MI_*CDH1*	22.25	19.50	14.13	42.80	40.75	11.71	46.42	44.75	20.91	28.83	24.75	9.09	b
	MI_*PAX1*	17.36	9.20	15.09	9.64	7.00	6.92	11.05	7.80	8.48	12.93	6.60	11.67	ns

Note: OC – ovarian cancer; BC-OC – ovarian cancer subsequent to breast cancer; P-value – statistical significance: ns – non significant difference between groups, single letters represent statistical significant difference (p<0.05) between groups: a – control and benign, b – control and OC, c – control and BC-OC, d – benign and OC, e – benign and BC-OC, f – OC and BC-OC.

Table S4: Sample size detrmination of tissue samples		
**Gene**	**Effect size**	**Sample size**
RASSF1		
CpG1	0.298	32
CpG2	0.266	40
CpG3	0.276	37
CpG4	0.313	29
CpG5	0.229	53
CpG6	0.348	24
PTEN		
CpG1	0.43	16
CpG2	0.36	23
CpG3	0.246	47
CpG4	0.286	35
CpG5	0.213	61
CDH1		
CpG1	0.487	13
CpG2	0.373	21
CpG3	0.254	44
CpG4	0.256	43
CpG5	0.343	25
PAX1		
CpG1	0.564	10
CpG2	0.394	19
CpG3	0.447	15
CpG4	0.359	23
CpG5	0.305	31
**Methylation indices**	**Effect size**	**Sample size**
MI *RASSF1*	0.298	32
MI *PTEN*	0.395	19
MI *CDH1*	0.361	22
MI_*PAX1*	0.449	15
Note: MI – methylation index		

Table S5. Sample size determination of plasma samples		
**Gene**	**Effect size**	**Sample size**
RASSF1		
CpG1	0.286	35
CpG2	0.322	28
CpG3	0.367	22
CpG4	0.305	31
CpG5	0.332	26
CpG6	0.346	24
PTEN		
CpG1	0.319	28
CpG2	0.121	187
CpG3	0.153	118
CpG4	0.1	275
CpG5	0.431	16
CDH1		
CpG1	0.392	19
CpG2	0.528	11
CpG3	0.483	13
CpG4	0.474	14
CpG5	0.571	10
PAX1		
CpG1	0.229	54
CpG2	0.339	25
CpG3	0.234	51
CpG4	0.209	64
CpG5	0.285	35
**Methylation indices**	**Effect size**	**Sample size**
MI *RASSF1*	0.3	32
MI *PTEN*	0.237	50
MI *CDH1*	0.531	11
MI_*PAX1*	0.263	41
Note: MI – methylation index		
